# Guiding Fibroblast Activation Using an RGD‐Mutated Heparin Binding II Fragment of Fibronectin for Gingival Titanium Integration

**DOI:** 10.1002/adhm.202203307

**Published:** 2023-05-10

**Authors:** Aina Heras‐Parets, Maria‐Pau Ginebra, Jose Maria Manero, Jordi Guillem‐Marti

**Affiliations:** ^1^ Biomaterials Biomechanics and Tissue Engineering group Department of Materials Science and Engineering Universitat Politècnica de Catalunya – BarcelonaTech (UPC) Av. Eduard Maristany 16 Barcelona 08930 Spain; ^2^ Barcelona Research Center in Multiscale Science and Engineering UPC Av. Eduard Maristany 16 Barcelona 08930 Spain; ^3^ Institute for Bioengineering of Catalonia (IBEC) Barcelona Institute of Science and Technology (BIST) Barcelona 08028 Spain

**Keywords:** fibroblast activation, fibronectin, growth factors, soft‐tissue integration, titanium

## Abstract

The formation of a biological seal around the neck of titanium (Ti) implants is critical for ensuring integration at the gingival site and for preventing bacterial colonization that may lead to periimplantitis. This process is guided by activated fibroblasts, named myofibroblasts, which secrete extracellular matrix (ECM) proteins and ECM‐degrading enzymes resolving the wound. However, in some cases, Ti is not able to attract and activate fibroblasts to a sufficient extent, which may compromise the success of the implant. Fibronectin (FN) is an ECM component found in wounds that is able to guide soft tissue healing through the adhesion of cells and attraction of growth factors (GFs). However, clinical use of FN functionalized Ti implants is problematic because FN is difficult to obtain, and is sensitive to degradation. Herein, functionalizing Ti with a modified recombinant heparin binding II (HBII) domain of FN, mutated to include an Arg‐Gly‐Asp (RGD) sequence for promoting both fibroblast adhesion and GF attraction, is aimed at. The HBII‐RGD domain is able to stimulate fibroblast adhesion, spreading, proliferation, migration, and activation to a greater extent than the native HBII, reaching values closer to those of full‐length FN suggesting that it might induce the formation of a biological sealing.

## Graphical abstract

In this work, the heparin binding II (HBII) fragment of fibronectin (FN) is mutated to include an Arg‐Gly‐Asp (RGD) sequence for improving cell adhesion. The new HBII‐RGD fragment has the ability to induce fibroblast adhesion, proliferation, activation, and migration similarly to full‐length FN when immobilized on titanium. Results suggest that HBII‐RGD can be used for the biological sealing of dental implants.

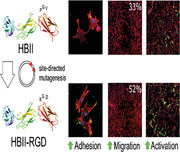

## 1 Introduction

The use of dental implants for the replacement of missing tooth has dramatically increased during the last years and is expected to rise according to the increment in life expectancy. However, there are still some cases that end with the implant failure mainly because of an inadequate interaction of the dental implant with the surrounding tissues.^[^ ^1^ ^]^ Dental integration success mainly relies on a complete interaction between the implant and bone (osseointegration), but the formation of a biological sealing at the gingival site (fibrointegration) is of paramount importance for the final outcome. This soft‐tissue integration is mediated by gingival cells, mostly fibroblasts, and is crucial for supporting peri‐implant tissues, improving aesthetics, preserving crestal bone levels, and particularly preventing bacterial colonization by microorganisms that may lead to periimplantitis, one of the main threats to the long‐term survival of dental implants.^[^ ^2^ ^]^

Among the different materials available for dental implants, titanium (Ti) is the most widely used due to its excellent biocompatibility and good mechanical properties.^[^ ^3^ ^]^ However, its lack of bioactivity has been associated to inadequate tissue interaction and consequently implant failure. To overcome this limitation, several efforts have been concentrated on modifying the Ti surface to promote soft tissue integration without changing its bulk properties.^[^ ^4^, ^5^, ^6^ ^]^ In this regard, one strategy is the functionalization of Ti surface with molecules that mimic the extracellular matrix (ECM), an intricate network composed of an array of macromolecules secreted by cells. The ECM not only provides structural and mechanical support for tissues and cells, but is also able to bind, store, and release at the appropriate amounts several molecules from the extracellular fluids, including growth factors (GFs).^[^ ^7^, ^8^ ^]^

GFs are proteins secreted by cells that play significant roles in cell survival, migration, proliferation, and differentiation. Hence, the use of GFs as therapeutic agents is a promising strategy for regenerative medicine and tissue engineering applications.^[^ ^9^ ^]^ However, the clinical use of GFs has been hampered mainly due to their short half‐lives upon injection/implantation, associated with their sensitivity to proteolytic degradation. This drawback implies the necessity of using several injections and/or higher doses than the physiological concentrations in order to present effective levels at the injured site, resulting in high costs and undesired side effects. Several biomaterial‐based approaches have been explored to address these issues, although most of them are based on the functionalization of scaffolds with GFs, which still require the use of exogenous GFs and dealing with their delivery at the appropriate levels.

An alternative approach that also has a strong impact in regenerative medicine is to design biomaterials that once implanted are able to attract specific endogenous GFs. To date, most of the ECM derived molecules used for this purpose have however promiscuous affinity for GFs. This is the case, for instance, of fibronectin (FN), an ECM glycoprotein that binds to several GFs with different affinities through its heparin binding II (HBII) domain.^[^ ^10^ ^]^ FN is found in all tissues of the body, and is especially present immediately following trauma, guiding the assembly of other ECM components and regulating adhesion, proliferation, migration, and differentiation of cells.^[^ ^11^ ^]^ Since FN plays significant roles in soft tissue regeneration, several studies have used it on Ti for inducing fibrointegration.^[^ ^12^, ^13^, ^14^ ^]^ However, clinical use of full‐length FN is challenging mainly because of the difficulty to purify it from human plasma and its sensitivity to proteolytic degradation.^[^ ^15^ ^]^ As an alternative, short FN recombinant fragments can be used. For instance, combination of the cell adhesion site (CAS) domain of FN, which contains the cell adhesive Arg‐Gly‐Asp (RGD) motif, with the aforementioned HBII domain has resulted in promising results, for both wound and bone applications.^[^ ^16^ ^]^ Nonetheless, controlling the appropriate distribution of both fragments at the surface is difficult to achieve,^[^ ^17^ ^]^ while using fused fragments increases the probability of proteolytic degradation.

In this context, in a previous study, we introduced a mutation in the HBII domain of FN to include an RGD sequence. Apart from inducing better cell adhesion and spreading in human mesenchymal stem cells compared to the native HBII, the novel mutated fragment notably increased its affinity to transforming growth factor beta (TGF‐*β*) by more than 10‐fold.^[^ ^18^ ^]^ TGF‐*β* is a key GF in wound healing, as it promotes the transition of fibroblasts into myofibroblasts, cell effectors during wound closure.^[^ ^19^ ^]^ Functionalization of Ti with TGF‐*β* to promote soft tissue integration has not yet been extensively explored, although if TGF‐*β* is not present at the appropriate concentration it might induce a fibrotic reaction ending with implant failure.^[^ ^20^ ^]^ Therefore, the use of the mutated HBII as endogenous TGF‐*β* attracting and releasing coating could be a promising strategy for inducing soft tissue integration of Ti at the gingival site.

The goal of the present study was to generate a surface able to induce the fibrointegration of Ti, by immobilizing a modified HBII fragment from FN that includes an RGD sequence to stimulate fibroblast adhesion and activation at the same time. The effects of the novel RGD‐mutated HBII fragment on the behavior of human fibroblasts were evaluated by measuring adhesion, proliferation, activation, and migration. Results were compared to the native HBII fragment and full‐length FN. Combined with our previous results observed on osteointegration, in the present study we wanted to demonstrate that a single RGD‐mutated HBII coating on Ti should be able to stimulate osteointegration or fibrointegration depending on the area that the dental implant will be contacting.

## 2 Results and Discussion

In the present study, Ti discs were polished to remove the effect of roughness, which has been observed to regulate the adhesion and activation of gingival fibroblasts.^[^ ^21^ ^]^ After polishing the discs, the average roughness of the surface measured by interferometry was Ra = 42.3 ± 0.6 nm showing a mirror‐like structure.

In a previous work we genetically modified the HBII fragment of FN to display an RGD sequence with the aim to not only attract GFs at Ti surface but also to attract cells through the interaction with integrins.^[^ ^18^ ^]^ In that study, we demonstrated the interaction of the novel HBII‐RGD protein with integrin *α*5*β*1, the most expressed integrin in mesenchymal stem cells. This was corroborated by an increase in cell spreading compared to native HBII, in addition to the generation of focal adhesions and to some extent actin cytoskeletal assembly. We have previously demonstrated that the covalent functionalization of Ti with silanes is an excellent method for coating FN fragments (**Table** **1**), obtaining a thickness consistent with a monolayer.^[^ ^22^, ^23^ ^]^ The increase in N 1s and C 1s, attributed to the presence of peptide bonds and amino acid side chains, demonstrates the existence of a protein coating when comparing the functionalized samples with bare Ti. In addition, the decrease in Ti 2p, Si 2p, and O 1s is also attributed to the presence of a protein monolayer. To demonstrate the durability of the covalently attached coatings, in the present study, we further studied the stability of the coatings in time. To this end, we stored the coated samples for 6 months under vacuum conditions and afterward we analyzed the chemical composition by X‐ray photoelectron spectroscopy (XPS). As shown in Table 1, similar values were obtained for all the conditions compared to the results obtained in the immediately prepared samples, suggesting that the coatings are still covalently attached to the Ti surface. In order to further analyze the stability of the coatings, we performed water contact angle measurements to demonstrate the presence of FN and FN fragments. The immobilization of FN onto Ti resulted in a significant increase in the value of water contact angle (Figure S1, Supporting Information). This behavior has been previously observed by other authors, and might be attributed to the hydrophobic character of FN.^[^ ^24^ ^]^ In contrast, the immobilization of the HBII fragment induced a more hydrophilic surface, while addition of the HBII‐RGD fragment showed intermediate values. Noteworthy, the water contact angle decreased in all samples stored for 6 months compared to samples immediately prepared, which could be indicating that proteins are changing their conformations or denaturing. To further confirm this, we analyzed the bioactivity of the samples stored for 6 months by culturing human foreskin fibroblasts (hFFs) and comparing their spreading with hFFs cultured on samples immediately prepared (Figure S2, Supporting Information). In general, we observed similar trends on aged samples compared to new samples when comparing the different coatings. Nonetheless, cells presented statistically significant lower area values when comparing the same coating stored for 6 months with the immediately prepared coating. This behavior was more dramatic for the FN coating, where cells presented an elongated morphology on samples stored for 6 months compared to a completely spread morphology on samples immediately prepared. Hence, these results suggested that although the coatings seem to be stable in time, the proteins might suffer conformational changes or degradation and lose bioactivity. A more extensive experiment with additional time points could be useful for determining the expiration date of the coatings under vacuum conditions, indicating for how long they could be stored before implantation.

**Table 1:** Surface elemental atomic composition (%) and thickness of immediately prepared coatings (as prepared) and stored in vacuum conditions for 6 months (aged 6 months)

	As prepared* ^)^						Aged 6 months					
	C 1s	O 1s	N 1s	Ti 2p	Si 2p	Thickness [nm]	C 1s	O 1s	N 1s	Ti 2p	Si 2p	Thickness [nm]
FN	57.46 ± 0.64	24.36 ± 1.12	10.82 ± 1.06	1.02 ± 0.11	6.34 ± 0.34	7.06 ± 0.84	54.95 ± 0.33	25.37 ± 1.23	9.71 ± 0.59	1.63 ± 0.07	8.36 ± 0.25	6.43 ± 0.52
HBII	46.45 ± 1.48	32.85 ± 1.22	10.26 ± 0.53	6.78 ± 0.72	3.66 ± 0.08	7.49 ± 0.13	64.05 ± 2.09	19.07 ± 1.18	12.36 ± 1.67	0.70 ± 0.30	3.82 ± 1.31	9.81 ± 1.46
HBII‐RGD	60.47 ± 0.05	20.43 ± 0.17	14.41 ± 0.29	0.79 ± 0.06	3.89 ± 0.13	5.08 ± 0.25	59.89 ± 0.48	21.60 ± 1.02	12.12 ± 1.13	1.11 ± 0.10	5.29 ± 0.28	7.29 ± 0.62
Ti	22.96 ± 1.87	56.25 ± 1.45	0.76 ± 0.19	19.57 ± 0.15	0.49 ± 0.08	‐	26.33 ± 2.34	52.60 ± 0.88	0.92 ± 0.01	19.92 ± 1.48	0.24 ± 0.02	‐

*) As prepared values were obtained in previous studies except for FN.^[^ ^17^, ^18^ ^]^

The oral cavity is a very dynamic environment presenting pH fluctuations maintained near the physiological pH by saliva, although in a slightly acidic range (from 6.2 to 7.6). Then, to test the potential effect of pH on the coated implants during surgical intervention, we incubated the samples at different pH (6.0, 6.5, 7.0, and 7.4) for 24 h or 48 h before seeding the cells. In general, no differences were observed in terms of area or circularity for those cells cultured on samples incubated for 24 h in the different pH values compared to physiological pH, that is, 7.4 (Figure S3, Supporting Information). A slight decrease in cell area values was observed when cells were cultured on samples incubated for 48 h compared to cells cultured on samples incubated for 24 h, regardless of the pH of the medium (Figure S4, Supporting Information). Hence, we hypothesize that the deposition of cell culture components in static conditions for 48 h might have a detrimental effect on cell adhesion. This hypothesis, however, should be demonstrated in further experiments. It is important to mention that coated implants are expected to be in contact with the oral acidic environment for a short period of time before the gingival area contacts with the implant, afterward reaching a pH close to neutral. Hence, we expect the coating to be functional during this short period of time as demonstrated by the results obtained. Since we observed no major influence of pH on the functionality of the coated discs, all the next experiments were performed at pH 7.4.

Another important aspect is the mechanical stability of the coating, which should not be removed due to frictional forces during the surgical procedure. There is no test other than performing an in vivo study to demonstrate that the coating remains at the surface during the insertion of the implant into the bone. Further experiments will elucidate this question, although there is a number of in vivo works in the literature demonstrating that either FN or FN fragments functionalized on Ti are still functional upon implantation.^[^ ^14^, ^16^, ^25^ ^]^

**Figure 1:**
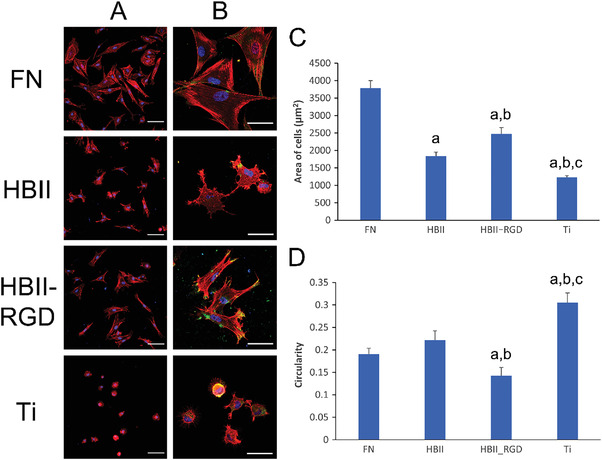
Cell spreading on the functionalized Ti surfaces. Representative images of hFFs after 3 h of adhesion on the different functionalized surfaces acquired at A) low magnification and B) high magnification (B). Scale bar denotes 50 and 20 µm, respectively. C) Calculated area and D) circularity of hFFs after 3 h of adhesion. Letter a indicates statistically significant differences compared to FN, letter b indicates statistically significant differences compared to HBII and letter *c* indicates statistically significant differences compared to HBII‐RGD (*p* < 0.05).

In the present study, we wanted to evaluate if the RGD‐mutated HBII fragment was able to induce fibroblast adhesion and spreading, and afterward stimulate fibroblast activation. Integrin *α*5*β*1 is one of the main integrin receptors of fibroblasts, allowing them to interact with the ECM through FN, which is found in high amounts during wound healing.^[^ ^26^ ^]^ Hence, integrin *α*5*β*1 plays a critical role in vivo during fibroblast invasion into the wound clot.^[^ ^27^ ^]^ Cells were completely spread on FN‐functionalized Ti surfaces, presenting well‐developed actin stress fibers and focal adhesions formation (**Figure** **1**A,B). In contrast, cells cultured on bare Ti showed a round morphology, with no sign of actin filaments and diffuse vinculin staining. Cells cultured in presence of the HBII domain presented an intermediate state, showing filopodia extensions and some vinculin spots at the edges of cells although actin stress fibers were barely observed. Noteworthy, addition of RGD to the HBII domain (HBII‐RGD) stimulated the extension of cells on Ti, and the formation of long actin stress fibers and focal adhesions, mostly at the edges of cells.

The calculated area of the cells was in accordance with the morphology, observing an intermediate value between FN and bare Ti for HBII and a slightly higher value for HBII‐RGD compared to HBII (Figure 1C). It is important to highlight that cells cultured on HBII‐RGD exhibited a more elongated morphology compared to FN‐coated surfaces. In fact, circularity values were the lowest for those cells cultured on HBII‐RGD, while for the other conditions the circularity values followed an inverse behavior compared to the area of cells (Figure 1D). The spindle‐shaped morphology observed in fibroblasts adhered to HBII‐RGD functionalized surfaces might be compatible with their activation into myofibroblast, which usually exhibit a contractile phenotype.^[^ ^28^ ^]^ Another important feature exhibited by activated fibroblasts is the presence of elongated nuclei, which can be observed in some of the cells cultured on HBII‐RGD surfaces (Figure 1A,B). Elongated nuclei have been related to increased histone acetylation, gene expression, and cellular activity,^[^ ^29^ ^]^ which mediate fibroblast proliferation and activation into myofibroblasts.^[^ ^30^ ^]^

**Figure 2:**
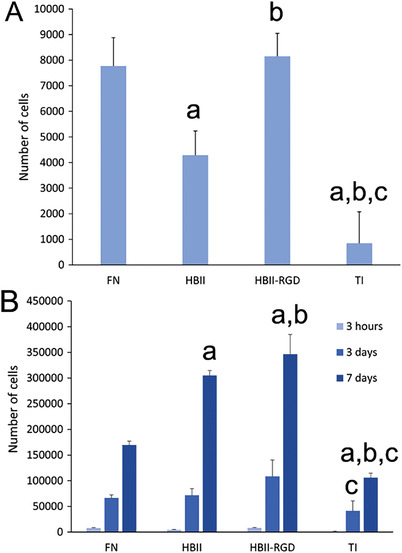
Number of cells on the functionalized Ti surfaces measured by LDH activity. A) Adhesion and B) proliferation of hFFs on the different functionalized and non‐functionalized Ti discs. At each time point, letter a indicates significant differences compared to FN, letter b indicates significant differences compared to HBII and letter c indicates significant differences compared to RGD‐mutated HBII (HBII‐RGD) (*p* < 0.05).

In general, functionalization of Ti with FN or FN fragments dramatically increased the number of adhered cells compared to plain Ti (**Figure** **2**A). FN is one of the main ECM proteins that fibroblasts interact with, hence it is not surprising that more than 80% of the cultured hFFs were adhered to the FN‐functionalized surfaces at the culture time used in the present study. In general, attachment of fibroblasts to FN is directed via the interaction of cell surface integrin *α*5*β*1 and the CAS domain of FN, which contains an RGD sequence and a Pro‐His‐Ser‐Arg‐Asn (PHSRN) synergy sequence.^[^ ^31^ ^]^ But there are also other domains that can contribute to fibroblast adhesion and spreading to FN. For instance, the HBII domain is known to interact with syndecan‐4, inducing focal adhesion formation and maturation only when CAS is present.^[^ ^32^, ^33^ ^]^ Also, the HBII domain contains several sequences able to interact with integrin *α*4*β*1,^[^ ^34^ ^]^ although this integrin binds poorly to HBII and to purified plasma FN.^[^ ^35^ ^]^ Complete binding to this integrin only occurs when a cryptic sequence located at the alternatively spliced type III connecting segment (IIICS) of FN, a fragment contiguous to HBII, is exposed due to proteolysis during tissue remodeling.^[^ ^36^, ^37^ ^]^ All of these domains are present in FN and are responsible for the adhesion and spreading levels found in the present study. In contrast, cells cultured on HBII‐functionalized surfaces can only interact through syndecan‐4 and integrin *α*4*β*1, which seemed to be sufficient to attract hFFs at the surface but at considerably lower levels compared to full‐length FN, and not enough for stimulating focal adhesion formation (Figure 1A,B). The introduction of RGD in the HBII domain was capable of doubling the number of hFFs attached to Ti compared to the native HBII, recovering the values obtained with full‐length FN coated surfaces (Figure 2A).

**Figure 3:**
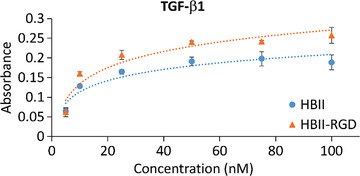
Interaction of native and mutated HBII fragments with TGF‐*β*1. Dose response curves for TGF‐*β*1 incubated on functionalized Ti samples and detected by ELISA.

The ability of fibroblasts to proliferate on the different functionalized Ti surfaces was analyzed after culturing hFFs for 3 and 7 days, until they reached confluence. Interestingly, the surfaces functionalized with HBII and HBII‐RGD registered a significant increase in the number of cells after 7 days of culture compared to FN‐coated surfaces, which in turn presented higher numbers than plain Ti (Figure 2B). This increase in cell proliferation may be attributed to the GF binding capacity of the HBII domain of FN.^[^ ^10^ ^]^ Among the different GFs that the HBII domain can bind to, TGF‐*β* has been reported to enhance fibroblast proliferation by induction of bFGF‐2 expression.^[^ ^38^ ^]^ Since FN inherently contains an HBII domain, differences in cell proliferation between FN‐ and HBII‐coated surfaces can be explained by the amount of HBII domains on the functionalized surfaces. The HBII domain is considerably small compared to full‐length FN, which implies that surfaces containing the HBII domain will present more GF‐attracting molecules compared to FN‐coated surfaces. Noteworthy, fibroblasts cultured on HBII‐RGD exhibited higher proliferation rates compared to native HBII (Figure 2B). This result might be explained by two reasons, or the combination of both: i) first, the presence of RGD, which has been described above to interact with integrin *α*5*β*1, can be leading to an orchestrated synergistic action of integrin and TGF‐*β* signaling, which induce focal adhesion formation and ultimately proliferation;^[^ ^39^ ^]^ ii) and second, an increased affinity of HBII‐RGD for TGF‐*β* compared to the native HBII domain. In fact, in a previous study, we observed an increase in TGF‐*β* binding by quartz crystal microbalance in HBII‐RGD due to structural or polarity changes attributed to the presence of RGD.^[^ ^18^ ^]^ In the present study, we confirmed this increase in TGF‐*β* binding affinity by enzyme‐linked immunosorbent assay (ELISA), a more reliable method that allowed us to obtain a dose‐response curve (**Figure** **3**). Interestingly, the growth rate of the obtained curve was higher in RGD‐mutated HBII domain compared to the native HBII, indicating a better affinity for TGF‐*β*.

**Figure 4:**
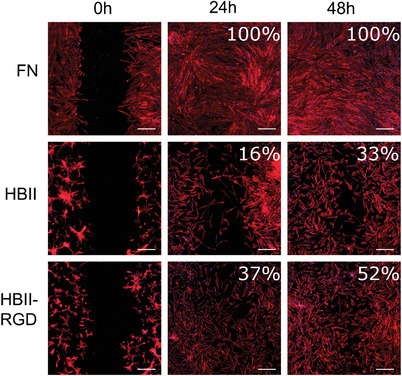
Migration of fibroblasts on the different functionalized surfaces. Images were acquired at 0, 24, and 48 h and gap closure percentages are displayed in each image. Scale bar denotes 200 µm.

We then evaluated the capacity of fibroblasts to migrate on the different functionalized Ti surfaces by using a silicone culture insert to create a cell‐free gap. Normally, FN is deposited in both the peri‐wound connective tissue and the provisional matrix generated during clotting, providing a conduit for fibroblasts to migrate into the wound space.^[^ ^40^ ^]^ There are three different FN domains required for such migration: the CAS, the HBII, and the IIICS domains.^[^ ^40^, ^41^ ^]^ Accordingly, in the present study hFFs were able to completely close the gap after 24 h when seeded on full‐length FN‐coated discs (**Figure** **4**), which obviously possesses the three domains. In contrast, cell migration was slower on HBII surfaces, with 16% gap coverage at 24 h and 33% at 48 h. Noteworthy, the presence of RGD in the mutated HBII promoted hFFs migration, resulting in an increased gap coverage at both 24 h (37% coverage) and 48 h (52% coverage) compared to the native HBII fragment. One limitation of this experiment however is the fact that cells were seeded without FBS overnight to allow cells to adhere to the FN fragments, resulting in a non‐completely covered surface because of a lower spreading and attachment compared to FN‐coated surfaces. Despite this limitation, the result allowed us to observe that the presence of RGD in the HBII domain is probably stimulating the expression of integrin *α*5*β*1 similar to the CAS domain,^[^ ^18^ ^]^ necessary for migration as mentioned above, although it was not enough for completely covering the gap. Regarding bare Ti, there were not enough cells attached to the surface at the beginning of the migration experiment, and therefore the acquisition of images and their quantification had no meaning (data not shown).

**Figure 5:**
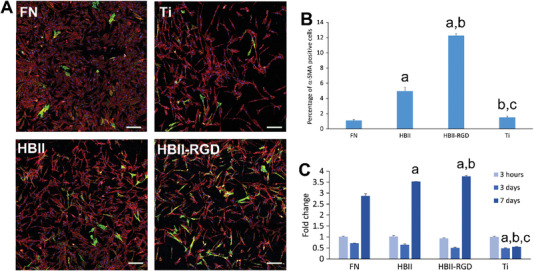
Fibroblast activation on functionalized surfaces. A) Representative immunofluorescence images of actin (red) and *α*‐SMA (green) of hFFs cultured on the different functionalized and non‐functionalized Ti discs. The scale bar denotes 200 µm. B) Quantification of the number of *α*‐SMA positive cells in the immunofluorescence images. C) Gene expression levels for *α*‐SMA of hFFs cultured on the different functionalized and non‐functionalized Ti discs. At each time point, letter a indicates significant differences compared to FN, letter b indicates significant differences compared to HBII and letter c indicates significant differences compared to RGD‐mutated HBII (HBII‐RGD) (*p* < 0.05).

After recruitment and proliferation, fibroblasts acquire a contractile phenotype in healing tissues. These activated fibroblasts are termed myofibroblasts and are characterized by the expression of alpha‐smooth muscle actin (*α*‐SMA).^[^ ^42^ ^]^ The transition from fibroblast to myofibroblast is a multi‐step process controlled by continuous chemical and mechanical changes in tissues under repair. Among the different stimuli, TGF‐*β* is a GF that strongly regulates the activation of fibroblasts via *α*‐SMA expression.^[^ ^43^ ^]^ Myofibroblasts were observed in higher percentages on the surfaces functionalized with HBII and particularly with HBII‐RGD domains in the present study (**Figure** **5**A,B). This may be explained by the aforementioned higher attraction of TGF‐*β* on these surfaces, which resulted also in higher gene expression of *α*‐SMA (Figure 5C).

**Figure 6:**
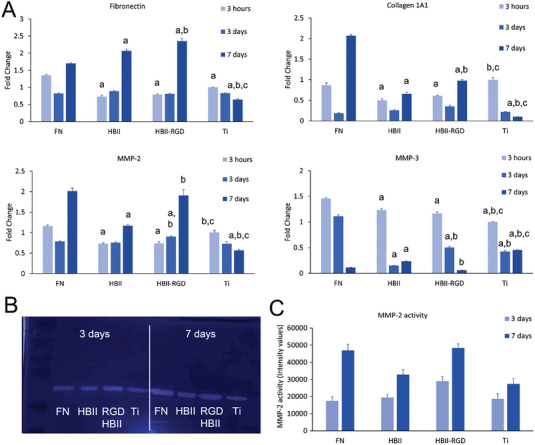
Fibroblast activation on functionalized surfaces. A) Gene expression levels for ECM proteins (FN and Collagen 1A1) and metalloproteases (MMP‐2 and MMP‐3) of hFFs cultured on the different functionalized and non‐functionalized Ti discs. B) Representative gelatin zymography image of conditioned media from hFFs cultured on the different functionalized and non‐functionalized Ti discs. C) Quantification of the MMP‐2 activity from the zymography. At each time point, letter a indicates significant differences compared to FN, letter b indicates significant differences compared to HBII and letter c indicates significant differences compared to RGD‐mutated HBII (HBII‐RGD) (*p* < 0.05).

The presence of myofibroblasts at the initial stages of wound healing is critical for the final wound closure, because myofibroblasts participate in the new ECM formation and remodeling by secreting numerous cytokines and GFs, ECM components, and ECM‐remodeling enzymes.^[^ ^44^ ^]^ Once activated, myofibroblasts secrete large amounts of ECM proteins, mainly FN and collagen. The gene expression of FN was increased after 7 days of culture on all the substrates functionalized with FN and FN fragments (**Figure** **6**A). The interaction of cells with FN through both integrin *α*5*β*1 and syndecan‐4, which bind to the CAS and the HBII domains respectively, are known to be important not only for cell adhesion but also for cell signaling.^[^ ^45^ ^]^ This activation is essential for an effective cell response to the provisional matrix, promoting FN expression, activation, and assembly into a fibrillary structure. However, after the initial response, the HBII fragment seems to be more important for stimulating FN expression. This behavior could be attributed again to the capacity of the HBII domain for attracting TGF‐*β*, which in turn induces the expression of FN.^[^ ^46^ ^]^ The higher affinity of the HBII‐RGD domain for TGF‐*β* could explain the higher FN expression values compared to the native HBII domain. This hypothesis however does not explain why cells cultured on FN fragments expressed less collagen 1A1 than cells cultured on FN (Figure 6A), although TGF‐*β* also induces collagen expression.^[^ ^47^ ^]^ In fact, collagen matrix deposition into the ECM is a process that requires fibrillar FN assembly.^[^ ^48^ ^]^ Therefore, cells do not start synthesizing collagen until a fibrillar FN matrix is formed, a situation that is probably achieved earlier in the FN functionalized surfaces because FN is already present from the beginning of the experiment. In contrast, cells cultured on FN fragments require time to synthesize FN before forming fibrillar structures, therefore expressing collagen at a later stage. Notably, the expression levels of collagen for cells cultured on HBII‐RGD were significantly higher compared to the native HBII domain, in accordance with the synthesis of FN.

After the initial synthesis and deposition of ECM components, myofibroblasts secrete ECM‐degrading molecules such as matrix metalloproteases (MMPs). MMPs are endopeptidases that cleave ECM proteins playing a central role in resolving the wound, and also in tissue turnover.^[^ ^49^, ^50^ ^]^ In general, an increase in the gene expression of MMP‐2 was observed in all the functionalized Ti surfaces compared to bare Ti from 3 to 7 days of culture (Figure 6A). Interestingly, the increase in MMP‐2 expression in those surfaces functionalized with HBII‐RGD was at similar levels compared to FN‐coated surfaces. One of the activities of MMP‐2 is to degrade collagen 1,^[^ ^49^ ^]^ hence this result may indicate that the deposited ECM would be more mature in HBII‐RGD compared to the native HBII. The activity of MMP‐2, analyzed by gelatin zymography, showed similar results, in line with the expression analysis (Figure 6B,C). Noteworthy, MMP‐9 activity was not observed in the gelatin zymography and the expression was not detected by RT‐qPCR (data not shown). MMP‐9 is another collagenase that has been found expressed in acute inflammation,^[^ ^51^ ^]^ indicating that none of the surfaces used in our study are inducing a pro‐inflammatory phenotype. In this regard, MMP‐3 has also been found related to acute inflammation.^[^ ^51^ ^]^ The gene expression of MMP‐3 decreased over time in our study in all the surfaces, especially in those functionalized with FN and FN fragments (Figure 6A) suggesting that the surfaces do not induce inflammation, which is known to be detrimental for the implant outcome.^[^ ^52^ ^]^ It is worth highlighting that MMP‐3 expression decreased more slowly in HBII‐RGD surfaces compared to HBII, following a similar trend to that induced by full‐length FN. Besides being related to inflammation, MMP‐3 participates in the degradation of FN and other ECM proteins.^[^ ^53^ ^]^ The slow decrease in the expression levels of MMP‐3 may indicate that HBII‐RGD surfaces stimulated the deposition of mature FN similar to the FN‐coated surfaces in line with what was observed for MMP‐2 expression and activity, while the HBII surfaces were not able to do so. This hypothesis however would require additional experiments.

## 3 Conclusion

A novel recombinant protein fragment obtained by mutating the HBII fragment of FN has demonstrated potential benefits for fibrointegration of Ti implants. The HBII‐RGD fragment improved fibroblast adhesion, spreading, proliferation, and migration compared to the native HBII domain. In addition, the capacity of HBII‐RGD to bind TGF‐*β* with more affinity allowed the novel protein to activate fibroblasts into myofibroblasts, secreting higher levels of ECM proteins and ECM‐degrading enzymes compared to HBII, showing similar values to FN‐coated surfaces. These results indicate that functionalization of Ti implants with HBII‐RGD domains could be an alternative to the use of full‐length FN‐coated implants, potentially forming a biological sealing that might favor the soft‐tissue integration of dental implants.

## 4 Experimental Section

### Titanium Samples

Discs of commercially pure, grade 2 Ti (10 mm diameter and 2 mm thickness) were ground with silicon carbide papers of increasing grit (800, 1200, and 2500; Struers, Spain) and mirror polished with 0.05 µm colloidal silica. Afterward, the discs were ultrasonically cleaned thrice for 5 min each with cyclohexane, isopropanol, deionized water, ethanol, and acetone.

### Synthesis and Mutation of the HBII Fragment

The HBII fragment of FN, spanning the 12th to 14th type III repeats, and the RGD‐mutated HBII fragment were produced following standard recombinant DNA methodologies, as previously described.^[^ ^17^, ^18^ ^]^ Briefly, the DNA sequence corresponding to the HBII fragment was inserted into a pGEX‐6‐P1 plasmid using restriction enzymes. DH5*α* cells (ThermoFisher Scientific, USA) were transformed and constructs were purified and sequenced. Correct plasmids were mutated to introduce an RGD motif in the 14th type III repeat using the Quickchange Lightning site‐directed mutagenesis kit (Agilent Technologies, USA) in two rounds. The mutated plasmid was inserted into DH5*α* cells and was purified and sequence verified. BL21 cells (New England BioLabs, UK) were transformed with native or mutated HBII containing plasmid and colonies were dynamically cultured in Luria Broth (Invitrogen, USA) supplemented with 100 µg mL^−1^ ampicillin at 37 °C and 225 rpm. Protein fabrication was induced with 1 mm IPTG during 4 h at 37 °C and 225 rpm. Then, bacteria were centrifuged and sonicated and proteins were purified using a GSTrap affinity column (GE Healthcare, UK). The GST‐tag was removed on‐column and the resulting purified proteins were checked by electrophoresis. Protein concentrations were quantified by Pierce Comassie (Bradford) Protein Assay kit (ThermoFisher Scientific) following the manufacturer's instructions.

### Titanium Functionalization

The molecules were covalently attached to Ti discs by silanization as described in previous studies.^[^ ^17^, ^18^, ^54^, ^55^ ^]^ Briefly, Ti discs were activated by oxygen plasma at 12 MHz for 10 min in a Femto low‐pressure plasma system (Diener Electronic, Germany). Activated samples were rapidly immersed in 0.08 m (3‐Aminopropyl)triethoxysilane (APTES) solution (Sigma Aldrich, USA) in anhydrous toluene at 70 °C for 1 h in a nitrogen atmosphere. After rinsing with different solvents, the discs were immersed in 7.5 mm solution of *N*‐succinimidyl‐3‐maleimidepropionate. Finally, the discs were coated with the native and mutated HBII fragments at 100 µg mL^−1^ in phosphate‐buffered saline (PBS). FN‐coated Ti discs and uncoated polished Ti discs were used as positive and negative controls, respectively.

### Physicochemical Characterization—Roughness

The roughness of polished Ti discs was measured using a white‐light optical profiling system (Wyko NT9300, Veeco Instruments, USA). An area of 736 × 480 µm was scanned in vertical scanning interferometry mode. Analysis of the data was performed with Wyko Vision 232 software (Veeco Instruments). A total of 3 different measurements were performed on randomly selected areas of 5 different polished Ti discs.

### Physicochemical Characterization—Contact Angle

The wettability of the different coated Ti samples was determined by sessile drop method using an Oca15 plus Contact Angle System (Dataphysics, Germany). Ultrapure distilled water (drop volume of 1 µL) was used as wetting liquid. A total of 5 measurements were performed at room temperature on randomly selected areas of 3 samples per each condition. In order to evaluate the stability of the coating, the water contact angle of the coated samples was also evaluated after sonicating the discs for 20 min.

### Physicochemical Characterization—Surface Chemical Composition

The surface chemical composition of bare Ti and coated Ti discs was analyzed by XPS. The spectra of C, O, N, S, and Ti elements were acquired with an XR50 Al anode source operating at 150 W in a Phoibos 150 analyzer and an MCD‐9 detector on an XPS system (SPECS Surface Nano Analysis, Germany). High resolution spectra were recorded at 0.1 eV steps after fixing the pass energy at 25 eV under ultra‐high vacuum conditions (7.5 × 10^−9^ mbar). All binding energies were referenced to the C 1s signal, which was calibrated at 284.4 eV. The thickness of the coatings was estimated by the attenuation of the XPS Ti signal according to previous works.^[^ ^56^, ^57^ ^]^

### TGF‐*β* Binding Capacity

The capacity of the native and the RGD‐mutated HBII fragments to bind TGF‐*β*1 was determined by a modified ELISA. Ti‐functionalized discs were rinsed thrice in 0.01% Tween‐20 in PBS (PBS‐T) and blocked with 1% bovine serum albumin (BSA) for 1 h. After rinsing with PBS‐T, the discs were immersed in increasing concentrations of TGF‐*β*1 (ranging from 0 to 100 nm; Peprotech, USA) for 2 h. Then, the discs were washed five times in PBS‐T and incubated with mouse anti‐TGF‐*β*1 (1:100; Santa Cruz Biotechnologies, USA) for 1 h. After five washes in PBS‐T, the discs were incubated with goat anti‐mouse immunoglobulins/horseradish peroxidase (1:2000; Agilent Dako, USA) for 1 h. Detection was performed by incubating the samples with 3,3',5,5'‐tetramethylbenzidine (TMB; Sigma Aldrich) substrate solution for 10 min and stopping the reaction with H_2_SO_4_. Absorbance was measured at 405 nm using a Synergy HTX multi‐mode reader (Bio‐Tek, USA).

### Fibroblast Behavior—Cell Culture

hFFs (Millipore, USA) were cultured in Dulbecco's Modified Eagle's medium supplemented with 10% fetal bovine serum (FBS), 20 mm HEPES, 50 U mL^−1^ penicillin, 50 µg mL^−1^ streptomycin and 2 mm L‐glutamine, all from ThermoFisher Scientific. Cells were maintained at 37 °C in a humidified atmosphere and 5% CO2.

### Fibroblast Behavior—Fibroblast Morphology and *α*‐SMA Staining

Cells were seeded in serum‐free conditions at a concentration of 20 000 cells per disc and allowed to adhere for 3 h. For the *α*‐SMA staining, the medium was replaced with complete medium, and cells were allowed to grow for 24 h. Cells were then fixed in 4% paraformaldehyde (PFA) for 20 min and permeabilized with 0.05% Triton X‐100, both in PBS. Next, cells were blocked with 1% BSA in PBS for 30 min and incubated for 1 h either with mouse anti‐vinculin (1:100; Sigma Aldrich) or mouse anti‐*α*‐SMA (1:500; ThermoFisher Scientific) antibodies for morphology or myofibroblast staining, respectively. Afterward, cells were incubated with Alexa Fluor 546 phalloidin (1:300; ThermoFisher Scientific) and Alexa Fluor 488 goat anti‐mouse antibody (1:1000; ThermoFisher Scientific) in the dark for 1 h. Cells were washed thrice with 20 mm glycine in PBS after each incubation step. Nuclei were counterstained with DAPI and discs were mounted in Mowiol 4–88 before visualizing in an LSM800 confocal laser scanning microscope (Carl Zeiss, Germany). Image analysis and calculations were performed using ImageJ software (https://imagej.nih.gov/ij/). At least five randomly selected images were used at 10× magnification for calculating the area and circularity of cells.

### Fibroblast Behavior—Coating Stability

The coated discs were incubated with serum‐free medium at different pH for 24 h or 48 h to analyze the potential effect of pH on the bioactivity of the coatings. The medium was adjusted to the desired pH using HCl. Experiments were conducted at pH 6.0, 6.5, 7.0, and 7.4 (being the last value of the pH of DMEM medium containing HEPES). After each specified time point, cells were seeded in serum‐free conditions at a concentration of 20 000 cells per disc and allowed to adhere for 3 h. Afterward, cells were fixed in PFA and stained with phalloidin and DAPI as mentioned above. Images were acquired with a LSM800 confocal laser scanning microscope (Carl Zeiss) and analyzed with ImageJ software. At least five images were acquired from randomly selected areas at 10× magnification for calculating the area and circularity of attached cells.

Coated samples maintained for 6 months under high‐vacuum conditions were also used for analyzing the potential effect of aging in the bioactivity of the coatings. Cells were directly seeded on these samples in serum‐free medium at a concentration of 20 000 cells per disc and allowed to adhere for 3 h. Staining, image acquisition, and image analysis were performed as described above.

### Fibroblast Behavior—Cell Adhesion and Proliferation

Cells were seeded at a concentration of 10 000 cells per disc in serum‐free conditions and allowed to adhere for 3 h. Afterward, medium was removed and cells were cultured for 7 days in a 10% serum‐containing medium changing the medium every other day. After each incubation period (3 h, 3 and 7 days), cells were lysed with 300 µL of mammalian protein extraction reagent (M‐PER; ThermoFisher Scientific). The number of cells was calculated by measuring the lactate dehydrogenase (LDH) activity using the Cytotoxicity Detection kitPLUS (LDH) (Roche Applied Science, USA). The absorbance values registered at 492 nm in a Synergy HTX multi‐mode reader (Bio‐Tek) were used to calculate cell numbers using a calibration curve consisting on decreasing numbers of cells.

### Fibroblast Behavior—Migration

Silicone culture inserts (Ibidi, Germany) were placed on top of functionalized Ti discs to generate a cell‐free gap. Then, cells were seeded in serum‐free conditions in both chambers of the culture insert at saturating concentrations and were allowed to adhere for 24 h. Next, the insert was removed, the medium was aspirated and non‐adherent cells were removed after extensively rinsing with PBS. The attached cells were incubated with 1% serum‐supplemented medium for 24 h and 48 h. Afterward, cells were fixed in 4% PFA and stained with Alexa Fluor 546 phalloidin and DAPI as described above. Images were acquired using an LSM800 confocal laser scanning microscope and the gap closure was calculated using ImageJ software.

### Fibroblast Behavior—Gene Expression

Cells were cultured in serum‐free conditions at a concentration of 20 000 cells per sample. After 3 h, the medium was removed and cells were cultured in 10% serum‐supplemented medium for 7 days. At each incubation time, (3 h, 3 and 7 days) total RNA was obtained using the RNeasy Mini kit (Qiagen, Germany) and quantified using a Take3 micro‐volume plate in a Synergy HTX multi‐mode reader (Bio‐Tek). Equal amounts of RNA (100 ng) were converted to cDNA using the Quantitect Reverse Transcription kit (Qiagen). Each specific gene was amplified by real time quantitative PCR (RT‐qPCR) using the QuantiFast SYBR Green PCR kit (Qiagen) and specific primers (**Table** **2**) designed in a previous study.^[^ ^58^ ^]^ The fold change of gene expression was obtained after normalizing results to *β*‐actin gene and to non‐coated Ti discs at 3 h following the formula described in that study.

**Table 2:** Primer sequences of the human genes amplified by RT‐qPCR

Gene name	Gene symbol	Primer sequences
Fibronectin	FN	GAACTATGATGCCGACCAGAA GGTTGTGCAGATTTCCTCGT
Collagen, type I, alpha 1	COL1A1	AGGTCCCCCTGGAAAGAA AATCCTCGAGCACCCTGA
Matrix metalloproteinase 2	MMP‐2	CGGTTTTCTCGAATCCATGA GGTATCCATCGCCATGCT
Matrix metalloproteinase 3	MMP‐3	GCAAGGACCTCGTTTTCATT CTCTTGGGTATCCAGCTCGT
Matrix metalloproteinase 9	MMP‐9	GAACCAATCTCACCGACAGG GCCACCCGAGTGTAACCATA
Actin, alpha 2, smooth muscle	*α*‐SMA	CTGTTCCAGCCATCCTTCAT TCATGATGCTGTTGTAGGTGGT
Actin, beta	ACTB	AGAGCTACGAGCTGCCTGAC CGTGGATGCCACAGGACT

### Fibroblast Behavior—Zymography

Cells at a concentration of 20 000 cells per sample were cultured in serum‐free medium for 3 h. Then, medium was removed and cells were cultured in 10% serum‐supplemented medium for 7 days. The day before each incubation period (3 and 7 days), medium was removed and cells were incubated in serum‐free medium for 24 h. Then, conditioned media were collected and quantified by Pierce Coomassie (Bradford) Protein Assay kit (ThermoFisher Scientific). Equal amounts of protein were loaded in 8% SDS‐PAGE co‐polymerized with gelatin (1.5 mg mL^−1^) under non‐reducing conditions (150 V, 4 °C). The gels were then washed with Triton X‐100 and incubated in substrate buffer for 22 h at 37 °C.^[^ ^59^ ^]^ Enzymatic activities were quantified by densitometric scanning using ImageJ.

### Statistical Analysis

Experiments were performed thrice with three replicates per each condition. Results are shown as mean values ± standard error of the mean. Statistically significant differences between groups (*p*‐value < 0.05) were analyzed using the Kruskal‐Wallis non‐parametric test followed by Mann–Whitney U test with Bonferroni correction using the Minitab software.

## Conflict of Interest

The authors declare no conflict of interest.

## Supporting information

Supporting Information

## Data Availability

The data that support the findings of this study are available from the corresponding author upon reasonable request.
