# Supplementary material for: Guiding Fibroblast Activation Using an RGD‐Mutated Heparin Binding II Fragment of Fibronectin for Gingival Titanium Integration
Source: Adv Healthc Mater. 2023 May 10;12(21):2203307. doi: 10.1002/adhm.202203307 (PMC11468578; doi:10.1002/adhm.202203307)
Supplement: Supplementary file 1 — Supporting Information [file ADHM-12-2203307-s001.pdf]

ADVANCED HEALTHCARE MATERIALS

Supporting Information

for *Adv. Healthcare Mater.*, DOI 10.1002/adhm.202203307

Guiding Fibroblast Activation Using an RGD-Mutated Heparin Binding II Fragment of Fibronectin for Gingival Titanium Integration

*Aina Heras-Parets, Maria-Pau Ginebra, Jose Maria Manero and Jordi Guillem-Marti**

Supporting Information

Guiding fibroblast activation using an RGD-mutated heparin binding II fragment of fibronectin for gingival titanium integration

Aina Heras-Parets, [a,b] *Maria-Pau Ginebra,* [a,b,c] *Jose Maria Manero,* [a,b] *and Jordi Guillem-Marti,**[a,b]

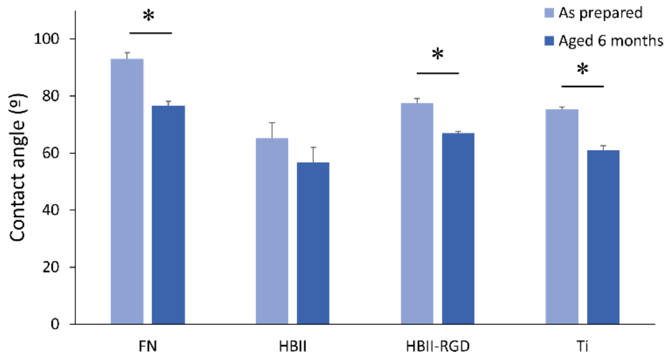

Figure S1. Water contact angle (º) values for the different functionalized surfaces immediately prepared (As prepared) or stored in vacuum conditions for 6 months (Aged 6 months). An * indicates statistically significant differences on aged samples compared to the same condition immediately prepared.

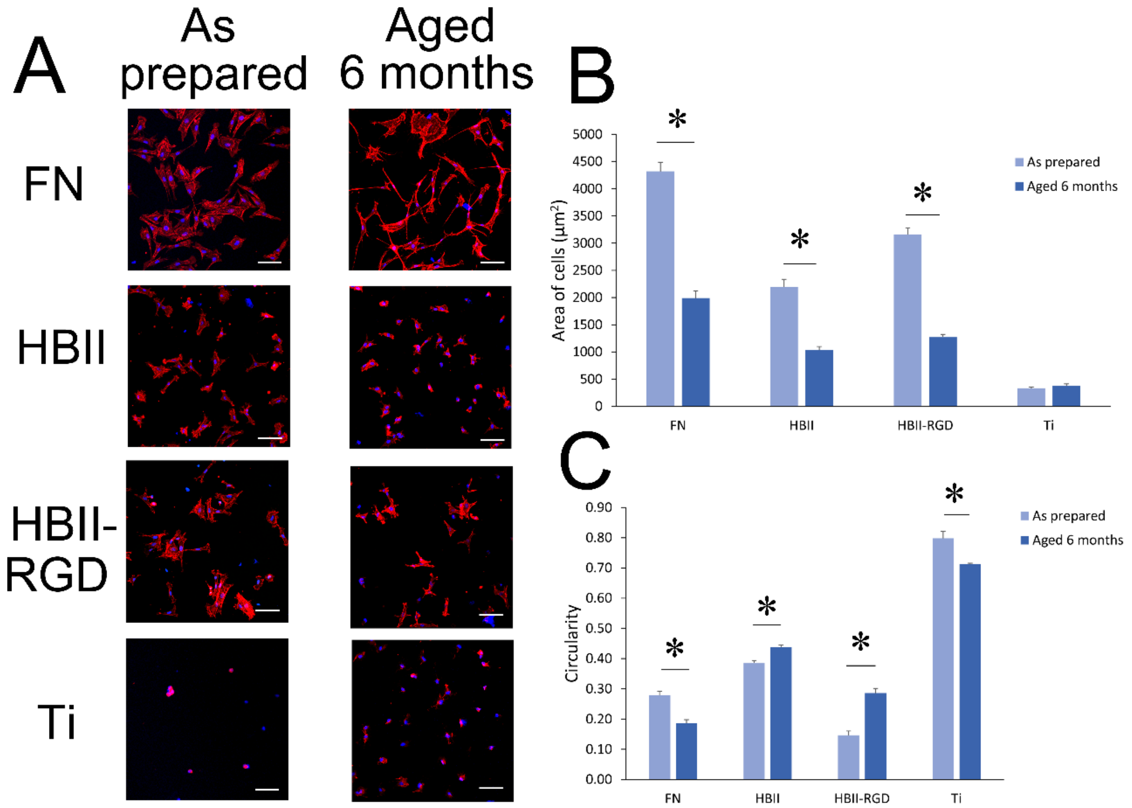

Figure S2. Effect of aging on the bioactivity of coated Ti after storing for 6 months in vacuum conditions. Representative images of hFFs after 3 h of adhesion (A) on the different functionalized surfaces prepared on the same day (As prepared) or stored for 6 months under vacuum conditions (Aged 6 months). Scale bar denotes 50 µm. Calculated area (B) and circularity (C) of hFFs. An * indicates statistically significant differences on aged samples compared to the same condition immediately prepared.

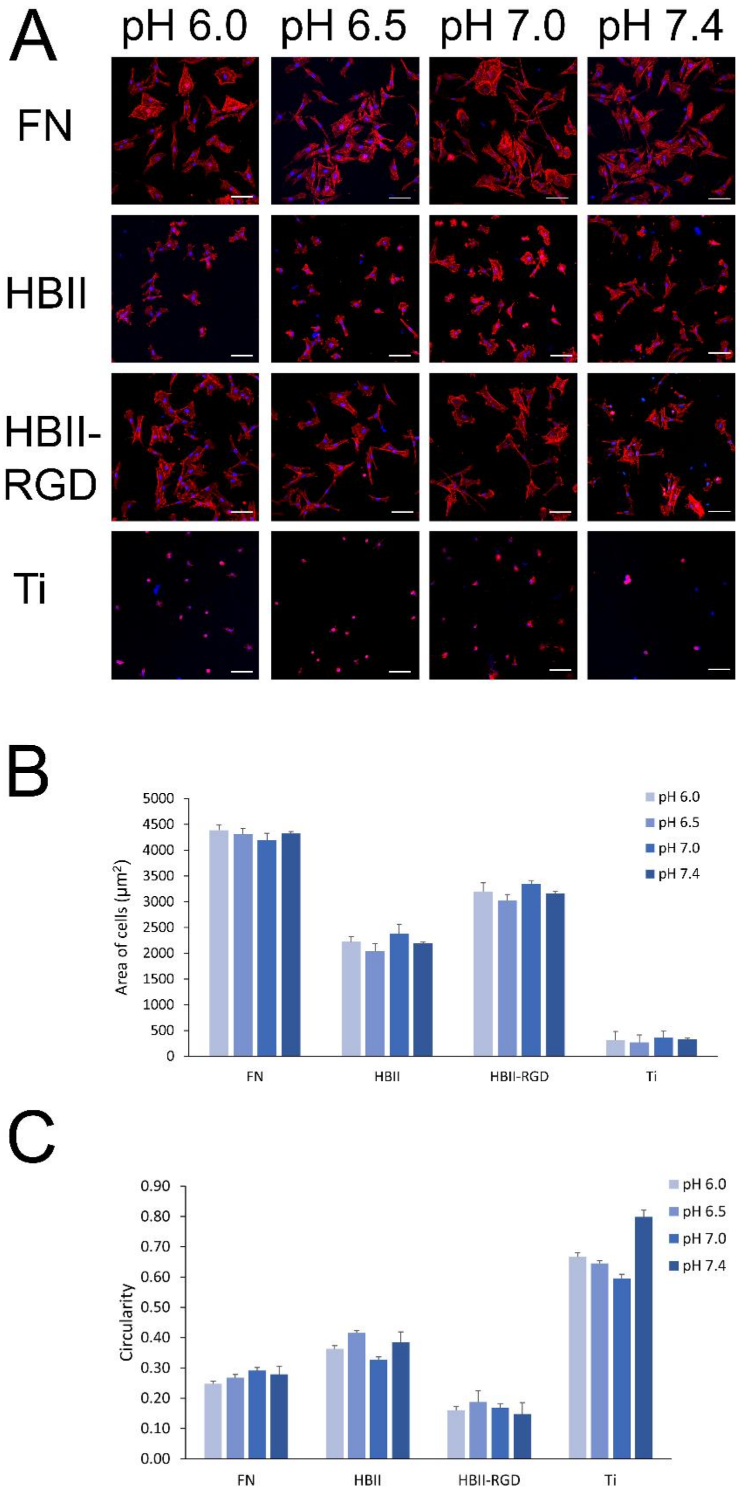

Figure S3. Effect of pH on the bioactivity of coated Ti after 24 h of incubation. Representative images of hFFs after 3 h of adhesion on the different functionalized surfaces pre-incubated at different pH conditions for 24 h (A). Calculated area (B) and circularity (C) of hFFs on the different pH conditions. Scale bar denotes 50 μm.

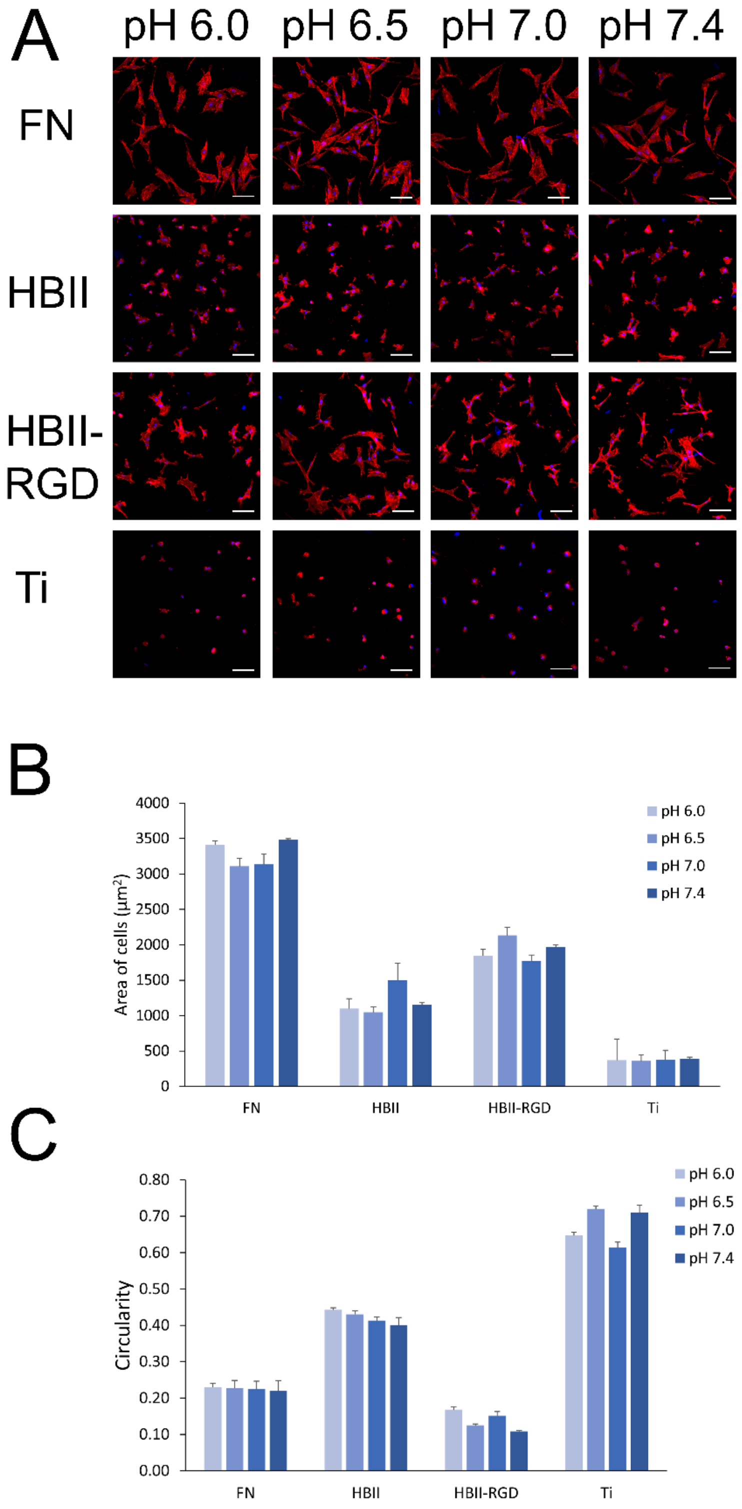

Figure S4. Effect of pH on the bioactivity of coated Ti after 48 h of incubation. Representative images of hFFs after 3 h of adhesion on the different functionalized surfaces pre-incubated at different pH conditions for 48 h (A). Calculated area (B) and circularity (C) of hFFs on the different pH conditions. Scale bar denotes 50 µm.
